# A new species of the genus *Arria* Stål, 1877 (Mantodea, Haaniidae) from China with notes on the tribe Arriini Giglio-Tos, 1919

**DOI:** 10.3897/zookeys.1025.56780

**Published:** 2021-03-18

**Authors:** Ying-jian Wang, Lin Yang, Fei Ye, Xiang-sheng Chen

**Affiliations:** 1 Institute of Entomology, Guizhou University, Guiyang, Guizhou, 550025, China Guizhou University Guiyang China; 2 The Provincial Special Key Laboratory for Development and Utilization of Insect Resources of Guizhou, Guizhou University, Guiyang, Guizhou, 550025, China Sun Yat-sen University Guangzhou China; 3 Guizhou Key Laboratory for Plant Pest Management of Mountainous Region, Guizhou University, Guiyang, Guizhou, 550025, China Guizhou University Guiyang China; 4 School of life sciences, Sun Yat-sen University, Guangzhou, Guangdong, 510275, China Sun Yat-sen University Guangzhou China

**Keywords:** Arriini, Cernomantodea, genitalia, Oriental Region, praying mantis, taxonomy

## Abstract

A new species of the praying mantis genus *Arria* Stål, *Arria pura***sp. nov.** from southwest China is described and illustrated. An overview, comparison, and distribution data of this tribe are given. A new synonym is created: *Sinomiopteryx yunnanensis* Xu, 2007 is a junior synonym of *Arria pallida* (Zhang, 1987). One new combination *Arria brevifrons* (Wang, 1991) **comb. nov.** (from *Sinomiopteryx* Tinkham), is proposed.

## Introduction

The tribe Arriini Giglio-Tos, 1919 (Mantodea, Haaniidae) comprises two genera, *Arria* Stål, 1877 and *Sinomiopteryx* Tinkham, 1937 (Schwarz and Roy 2019). The genus *Arria* was established by Stål (1877) based on the type species *A. cinctipes* Stål, 1877 from India. The other genus *Sinomiopteryx* was established by Tinkham (1937) based on the type species *S. grahami* Tinkham, 1937 from China. Recently, Schwarz and Roy (2018) proposed that *Palaeothespis* Tinkham, 1937 and *Pseudothespis* Mukherjee, 1995 are junior synonyms of *Arria*. So far, the number of species in the genus *Arria* and *Sinomiopteryx* has changed from six to eight and four to two, respectively, including the new species, new synonym and new combination (Xu 2007; Schwarz and Roy 2018; Otte et al. 2020).

The species of Arriini are quite similar in appearance and there are few previous descriptions and illustrations of male genitalia. In this paper, during a study of the external morphology and male genitalia of Arriini, we found that *Arria* and *Sinomiopteryx* can be easily distinguished by male genitalia and other valuable appearance features, which are also provided to help distinguish the two genera.

## Material and methods

Specimens were collected by sweeping net. The tip of the abdomen was separated and macerated in 10% water solution of KOH for about 12 hours, then washed in water and absolute ethanol. The terminalia were isolated into supra-anal and subgenital parts, the phallomeres were separated into left phallomere, ventral phallomere and right phallomere without overlapping, then all parts were preserved in glycerine in a microvial pinned with the specimen. Some wings were removed and mounted on a slide provided with the data of the specimen. External morphology and male genitalia were observed using a Leica M125 stereomicroscope. Photographs of phallomeres, terminalia and wings were taken with Nikon SMZ25, and photographs of the mounted specimens were taken with Canon EOS-70D digital camera with a Canon 100 mm Macro EF Lens or a Laowa 60 mm Macro Lens. Photo stacks were created using Helicon focus 6.0.18. Figures were processed with Adobe Photoshop CS6. Measurements were collected with the Keyence VHX-1000 system using the live measurement mode. Numeric patterns of the discoidal spines: The numbers (from left to right) represent the position (proximal to distal). Their value is representative of their respective length relation to the other spines with 1 being the shortest and 4 being the longest spine. Identical values represent equally long spines (Wieland 2013). L/W ratio of forewing means length to max width ratio of forewing. The classification system follows Schwarz and Roy (2019). Terminology and abbreviations follow Brannoch et al. (2017), for genitalia, we follow Schwarz and Roy (2019). Measurements follow Brannoch et al. (2017), but head length includes the labrum, foretibia length was from the base to the apex of the tibial spur, for apterous females the total length was from the vertex of the head to the posterior tip of the abdomen. The distribution map was created in SimpleMappr (Shorthouse 2010). Examined specimens were deposited in the following collections:

**NFU**Nanjing Forestry University, Nanjing, China;

**SEM** Shanghai Entomological Museum, CAS, Shanghai, China.

The type specimens of *Arria pura* sp. nov. are deposited in the Institute of Entomology, Guizhou University, Guiyang, Guizhou Province, China (IEGU).

### Abbreviations

**afa** phalloid apophysis;

**AvS** anteroventral spine;

**CuA** anterior Cubitus;

**DS** discoidal spine;

**fda** main posterior lobe of right phallomere;

**HT** holotype;

**L1** sclerite L1 of left phallomere;

**L2** sclerite L2 of left phallomere;

**L4** sclerite extending over most of the dorsal and ventral walls of left phallic;

**L4A** ventral sclerite of L4;

**L4B** dorsal sclerite of L4;

**loa** membranous lobe of left phallomere;

**paa** apical process of left phallomere;

**pda** primary distal process;

**pia** a process arising from the midlength to posterior right ventral wall of right phallomere, posterolateral to process pva;

**pva** a process arising from the midlength of the ventral wall of right phallomere, anteromesal to process pia;

**PvS** posteroventral spine;

**PT** paratype;

**R1A** dorsal sclerite of fda;

**R1B** sclerite on process pia and pva;

**R3** anteriorly extending sclerite of right phallomere;

**sdp** secondary distal process;

**sdpm** median secondary distal process;

**tl** terminal lobe of ventral phallomere in *Arria*.

## Checklist and distributions of species of Arriini

*A. brevifrons* (Wang, 1991), China (Zhejiang), comb. nov.

*A. cinctipes* Stål, 1877, India (Manipur).

*A. leigongshanensis*, (Ge & Chen, 2008), China (Guizhou, Yunnan).

*A. meghalayensis*, (Mukherjee, 1995), India (Meghalaya).

*A. oreophila*, (Tinkham, 1937), China (Sichuan).

*A. pallida*, (Zhang, 1987), China (Yunnan), *S. yunnanensis*, Xu 2007 is a junior synonym of *A. pallida*.

*A. pura* Wang & Chen, sp. nov., China (Guizhou).

*A. sticta* (Zhou & Shen, 1992), China (Guizhou, Hunan, Zhejiang).

*S. grahami* Tinkham, 1937, China (Sichuan).

*S. guangxiensis* Wang & Bi, 1991, China (Guangxi).

## Taxonomy

### Arriini

Taxon classificationAnimaliaMantodeaMantidae

Giglio-Tos, 1919

D6FF830F-4EED-5793-A60B-1A2596E71935

Arriini Giglio-Tos, 1919: 65; Schwarz and Roy 2019: 141.

#### Type genus.

*Arria* Stål, 1877.

#### Distribution.

China, India (Fig. 11).

### Arria

Taxon classificationAnimaliaMantodeaMantidae

Stål, 1877

AC7DE260-59FE-5833-A5A8-A3C8A1CF0CC7

Arria Stål, 1877: 46; Tinkham 1937: 497; Zhang 1987: 239; Zhou and Shen 1992: 62; Wang and Bi 1991: 125; Wang 1993: 114; Mukherjee and Ghosh 1995: 251; Ehrmann 2002: 72, 259, 298; Xu 2007: 244; Ge and Chen 2008: 53; Schwarz and Roy 2018: 456; Schwarz and Roy 2019: 141.

#### Type species.

*Arria cinctipes* Stål, 1877, original designation.

#### Diagnosis.

Body medium and slender (Fig. 7), female stronger than male. Head narrowly transverse with juxtaocular bulges; compound eyes broadly oval, prominent; ocelli large in male (Fig. 8), minute in female. Lower frons transverse, 3.3–4.2 times as wide as high. Antennae filiform, long in male, much shorter in female. Forefemur slender, with 4 discoidal, 10–13 anteroventral and 4 posteroventral spines; foretibia with 7–9 anteroventral and 4–7 posteroventral spines. Pronotum short with supracoxal dilatation well marked, lateral margins with small denticles in male and strongly tuberculate in female. Forewing narrow with narrowly rounded apex, CuA branches no less than 5, L/W ratio is 4.3–5.5; fore margin with widely spaced cilia, hindwing with pointed apex, vein M with brunet band near the tip; both pairs of wings fully developed and exceeding the end of abdomen in male (Fig. 9); female apterous. Sclerite L4A approximately rhomboidal, males with terminal lobe (tl) of ventral phallomere fused to vla, protruding as a truncate lobe, close to sdpm ventrad; sdpm short; right phallomere with large pia and ear-shaped pva. Styli close to each other (Fig. 4).

### Arria brevifrons

Taxon classificationAnimaliaMantodeaMantidae

(Wang & Bi, 1991) comb. nov.

4C556186-860C-5A99-B252-E297CA7281F6

Fig. 10A–D

Sinomiopteryx brevifrons Wang & Bi, 1991: 125; Wang 1993: 114.

#### Material examined.

***Holotype*,** 1♂, China: Zhejiang Prov., Qingyuan County, Baishanzu, 1100 m, 14.X.1963, Gen-tao Jin leg., ID: 0800123 (SEM).

#### Remarks.

This species was described and illustrated by Wang and Bi in 1991 based on one male specimen from Zhejiang. The ocelli are not closely grouped as in the males of *Sinomiopteryx*; forewings are narrow, L/W ratio is 4.8 with CuA_5_, hindwings with pointed apex and brunet band of near apex of vein M. All features fall into the range of *Arria*. The specimen has had the abdomen removed, but we did not find the genitalia. According to the spots on the forelegs, *A. brevifrons* can be distinguished from *A. pallida* and *A. sticta*.

### Arria cinctipes

Taxon classificationAnimaliaMantodeaMantidae

Stål, 1877

F8A756F5-2868-57BF-89F7-6ED8A8CE1C7B

Arria cinctipes Stål, 1877: 46; Schwarz and Roy 2018: 456.

### Arria leigongshanensis

Taxon classificationAnimaliaMantodeaMantidae

(Ge & Chen, 2008)

91D83325-A5DA-5457-885B-1B3509709052

Figs 4D–F , 9A , 10E–H

Palaeothespis leigongshanensis Ge & Chen, 2008: 53–58.

#### Material examined.

***Holotype*,** 1♂, China: Guizhou Prov., Leigong Mountain National Natural Reserve, 13.IX.2005, Qiong-Zhang Song leg.; ***Paratype***, 1♀, same locality as for holotype, 14.IX.2005, Zhi-Jie Wang leg. (IEGU).

### Arria meghalayensis

Taxon classificationAnimaliaMantodeaMantidae

(Mukherjee, 1995)

33B5CEA9-3C29-5028-A706-6920ADBD8C4D

Pseudothespis meghalayensis Mukherjee, 1995: 60; Schwarz and Roy 2018: 456.

### Arria oreophila

Taxon classificationAnimaliaMantodeaMantidae

(Tinkham, 1937)

91FCEE0C-32B4-5171-9BFF-3916C233618A

Palaeothespis oreophila Tinkham, 1937: 497–499; Svenson 2014: 55–56.

#### Remarks.

*Arria oreophila* was described and illustrated by Tinkham based on 1 male and 1 female from Sichuan, China. In Tinkham (1937), the foretibial consists of 13 anteroventral spines in males and 11 in females. But according to Svenson (2014), the anteroventral spines of the foretibia is R9/L8 in the male and 8 in the female. It falls into the range described for other species of *Arria*. *Arria oreophila* differs from others in both fore- and hindwings brownish, veins RP and M with two forks.

### Arria pallida

Taxon classificationAnimaliaMantodeaMantidae

(Zhang, 1987)

B9029870-0F8A-50E3-9F3E-8004A980556B

Figs 4G–I , 10I–L

Palaeothespis pallida Zhang, 1987: 239
Sinomiopteryx yunnanensis Xu, 2007: 244 syn. nov.

#### Material examined.

***Holotype*,** 1♂, China: Yunnan Prov., Lushui County, 10.X.1980, Guo-zhong Zhang leg. (NFU); 2♂♂, Yunnan Prov., Fenshuiling National Natural Reserve, 19.V.2015, Yun-fei Wu and Jia-jia Wang leg. (IEGU).

#### Remarks.

Xu (2007) described this species in *Sinomiopteryx* from Yunnan, China. The descriptions and illustrations for both external morphology and genitalia of *Sinomiopteryx yunnanensis* Xu, 2007 fall into the range presented for *Arria pallida* (Zhang, 1987), especially the significant differences of male genitalia between *Arria* and *Sinomiopteryx* (Figs 4A–L, 5A–F). Therefore, we consider *S. yunnanensis* Xu, 2007 as a junior synonym of *A. pallida* Zhang, 1987.

### Arria pura

Taxon classificationAnimaliaMantodeaMantidae

Wang & Chen sp. nov.

D4C2C1E6-107F-5758-A523-031690F62B7D

http://zoobank.org/12C0F813-0717-4B2D-81C7-1E713062E2AE

**Figure 1.**
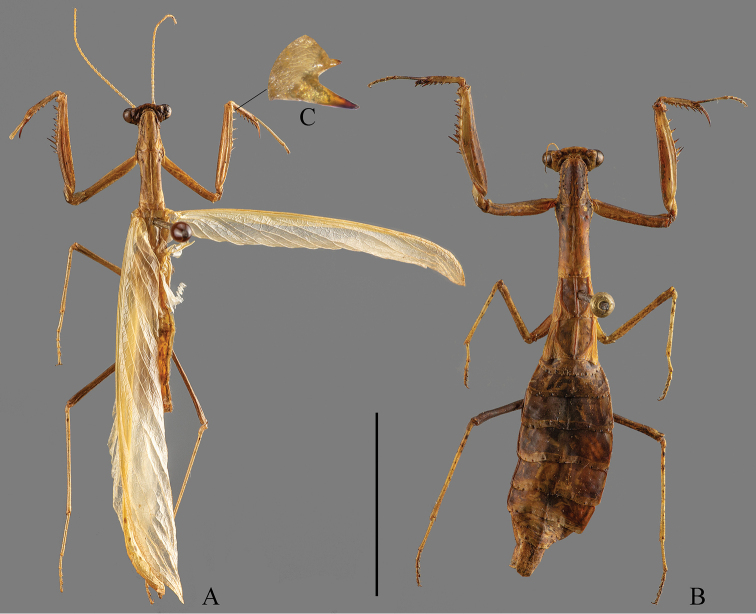
*Arria pura* sp. nov., dorsal habitus **A** male holotype **B** female paratype **C** posteroventral genicular spines of male forefemur (not to scale). Scale bar: 10 mm.

**Figure 2.**
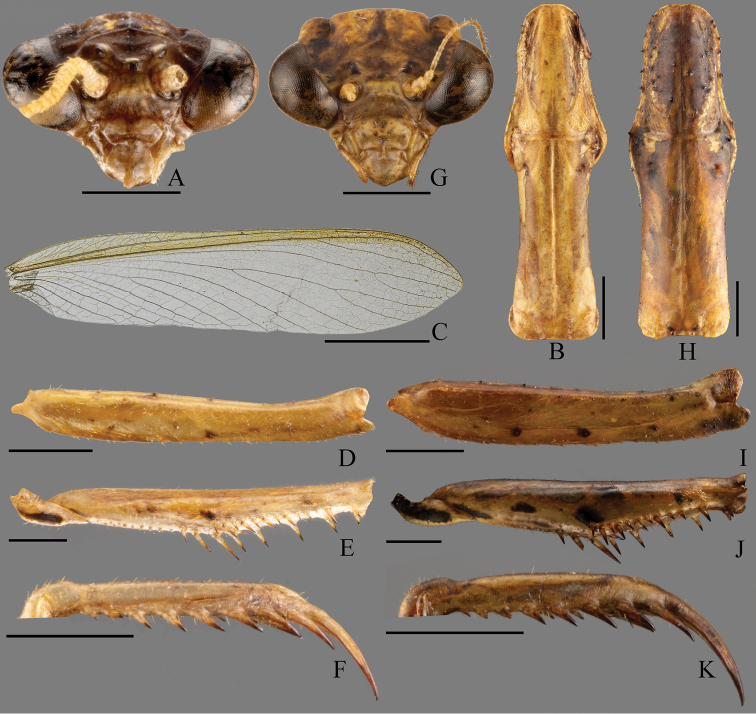
*Arria pura* sp. nov., details of morphology **A–F** male **G–K** female **A, G** head, frontal view **B, H** pronotum, dorsal view **C** forewing, dorsal view **D, I** forecoxa, ventral view **E, J** fore- trochanter and femur, ventral view **F, K** foretibia, ventral view. Scale bars: 5 mm (**C**); 1 mm (**A, B, D–K**).

**Figure 3.**
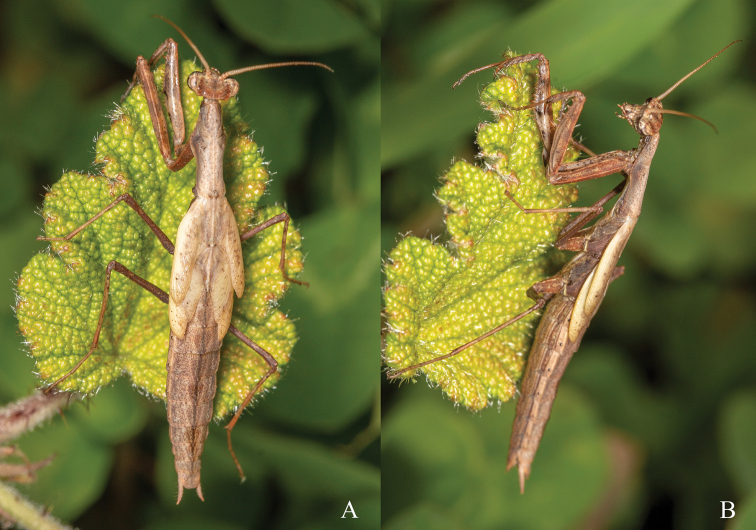
Male last instar nymph of *A. pura* sp. nov. holotype, life habitus **A** dorsal view **B** lateral view. Photograph by Ying-Jian Wang.

**Figure 4.**
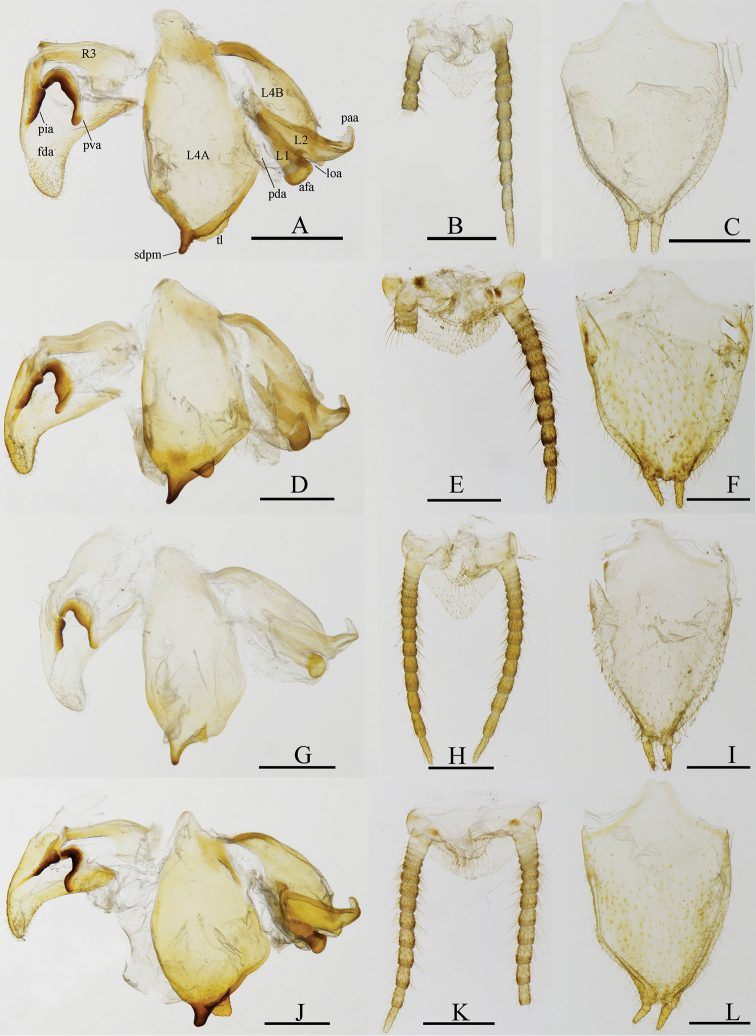
Male genitalia (ventral view) and terminalia (dorsal view) of *Arria* spp. **A–C***A. pura* sp. nov., holotype **D–F***A. leigongshanensis* (Ge & Chen, 2008), holotype **G–I***A. pallida* (Zhang, 1987) (IEGU: HAAP1) **J–L***A.* sp.2 (IEGU: HAASP2-1). Scale bars: 1 mm.

Figs 1 , 2 , 3 , 4A–C , 6

#### Type material.

***Holotype*:** ♂, China: Guizhou Province, Weining County, Jinzhong Town (26°42.34'N, 104°37.29'E), 2550 m, 17.VIII.2017, Ying-Jian Wang; ***Paratypes***: 1♀, same data and locality as holotype (IEGU), PT1; 1♀, Guizhou Province, Weining County, Xueshan Town (27°04.04'N, 104°06.68'E), 2450 m, 2.IX.2019, Feng-E Li, PT2.

#### Description.

Measurements are provided in Table 1.

**Table 1.** *Arria pura* sp. nov., measurements of type specimens, in mm.

Measured structure	HT (male)	PT1(female) #1	PT2(female)
Head width	2.6	3.2	3.1
Head length	1.8	2.5	2.3
Lower frons width	1.0	1.2	1.2
Lower frons length	0.4	0.5	0.4
Body length	26.3	23.0	21.9
Pronotum length	5.3	6.1	5.7
Prozone length	2.1	2.4	2.3
Metazone length	3.2	3.7	3.4
Pronotum max. width	1.7	2.1	2.0
Forewing length	21.6	–	–
Forewing max. width	4.0	–	–
Forecoxa length	4.3	4.7	4.5
Forefemur length	5.3	5.6	5.3
Forefemur max. width	0.7	1.0	1.0
Foretibia length	2.8	3.2	3.1
Foretarsus length	4.0	3.0	2.8
Mesocoxa length	1.5	1.6	1.8
Mesofemur length	4.4	4.0	3.8
Mesotibia length	3.2	3.0	2.9
Mesotarsus length	2.9	2.4	2.2
Metacoxa length	1.8	1.8	1.8
Metafemur length	5.2	4.7	4.5
Metatibia length	5.6	4.8	4.4
Metatarsus length	4.3	3.5	3.1
Subgenital plate length	1.7	1.9	1.6
Subgenital plate width	1.4	3.5	3.2

**Male.** Small, slender (Figs 1A, 3).

***Head*** (Fig. 2A). Triangular, about 1.4 times as wide as long. Vertex with pair of paramedian depressions, otherwise flat. Compound eyes oval and large, conspicuously projecting outside profile of head. Juxtaocular bulges present, extending to the dorsal edge of vertex. Ocelli large and elliptic, lateral paired ocelli larger. Antennae filiform, ciliated. Scapus cylinder-shaped, slightly depressed in the middle, approximate as length as width. Pedicellus almost as long as scapus yet narrower, goblet-space. Third antennomere about as long as pedicel. Fourth antennomere less than half of third length. Lower frons transverse, 4.2 times as wide as high, flat medially, with dorsal and lateral margins bordered by protruding ridge, ventral margin inconspicuous, dorsal margin obtuse-angled. Clypeus smooth, above of ventral margin with a transverse groove.

***Prothorax*** (Fig. 2B). Short, 3.2 times as long as wide, metazone 1.5 times as long as prozone, lateral margins with small and few setiferous denticulations, middle carina present but feeble in prozone, behind supracoxal sulcus with pair of depressions, posterior margin with pair of paramedian bulges. Prosternum with middle carina posteriad supracoxal sulcus.

***Cervix*.** No ventral cervical sclerite. Intercervical sclerite merged at middle, without torus intercervicalis. Postcervical plate and furcasternite flat.

***Metathorax*.** With cyclopean ear of DK type.

***Forelegs*.** Forecoxa longer than metazone with anterior lobes diverging, dorsal edge and ventral edge with 3–4 and 9–11 tubercules respectively, all tubercules with small seta (Fig. 2D); anterior and posterior surface of forecoxa mostly smooth. Forefemur (Fig. 2E) slender, with four posteroventral spines, almost as long as each other; four discoidal spines, numeric patterns: 1231, the fourth discoidal spines inclined toward apex strongly; eleven anteroventral spines, the resulting arrangement of the holotype being iIiIiIiIiiI; posteroventral genicular lobe with two spines arranged in a row and the small one near apex (Fig. 1C); anteroventral genicular lobe with a single spine; F = 4DS/11AvS/4PvS. Foretibia armament: six posteroventral spines, the apical two closes to each other; eight anteroventral spines, elongating distally; T = 8AvS/6PvS. Metatarsus 1.5 times as long as remaining tarsomeres combined.

***Middle and hind legs*.** Long, cursorial. Meso- and metafemur apically without genicular spine. Meso- and metatibia apically with two spines. Middle 2 to 5 tarsomeres combined 1.6 times as long as middle metatarsus. Hind metatarsus slightly longer than remaining tarsomeres combined.

***Wings*** (Fig. 2C). Fully developed, surpassing the end of abdomen. Forewing hyaline without spots, 5.4 times as long as width. Costal field reaches 4/5 of the forewing’s length. Stigma elongated, inconspicuous.

***Abdomen*.** Depressed dorsoventrally, coxosternite 9 (subgenital plate) (Fig. 4C) longer than wide, covered ventrally and laterally by numerous setae. Tergite 10 (supra-anal plate) (Fig. 4B) triangular. Cerci with approximate 12 cercomeres, difficult to distinguish from each other near the base, distal-most cercomere elongate.

**Figure 5.**
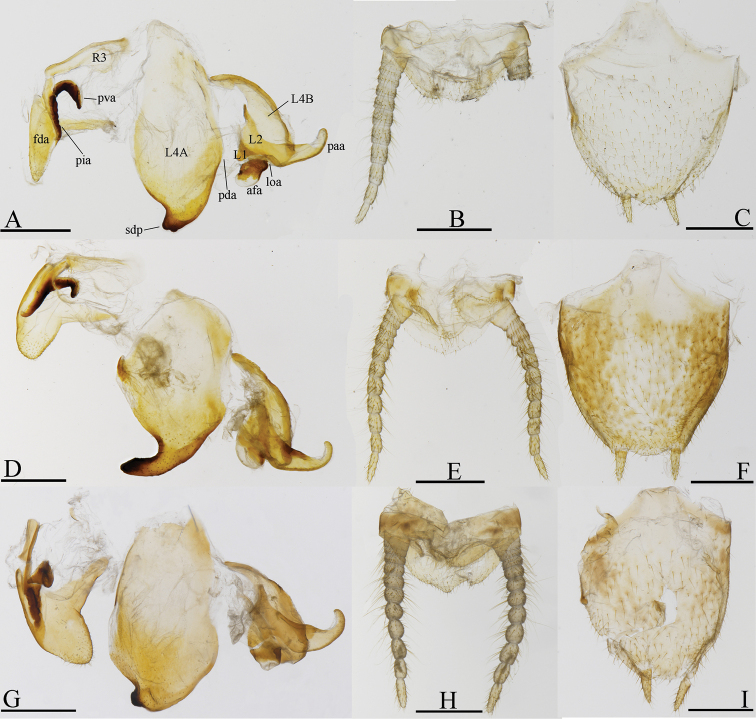
Male genitalia (ventral view) and terminalia (dorsal view) of *Sinomiopteryx* spp. **A–C***S. guangxiensis* Wang & Bi, 1991 (IEGU: HASG1) **D–F***S.* sp.1 (IEGU: HASSP1-1) **G–I***S.* sp.2 (IEGU: HASSP2-1). Scale bars: 1 mm.

***Male genitalia*** (Fig. 4A). Sclerite L4A approximately rhomboidal, with strongly transverse terminal lobe (tl) on right side of distal process in ventral view. Distal process inclined dorsolaterally, at an angle of 100° relative to the plane of L4A. Sclerite L4B winebottle-shaped, slightly curved near base. Sclerite L2 with apical process paa strongly curved, almost parallel to L4B dorsally. Sclerite L1 elongated, afa sclerotized, covered by numerous small granules apically. Fda of right phallomere sclerotized by sclerite R1A densely covered by long setae. Sclerite R1A more or less triangular. Sclerite R3 drumstick-shaped; sclerotized processes very developed. Sclerite R1B with pia well sclerotized, process pva large, ear-shaped.

***Coloration*.** General color brown. Vertex brown; juxtaocular bulges fuscous. Ocelli hyaline. Foretrochanter black ventrally, base anteroventral of forefemur black as well as base of tibial spur groove (Fig. 2E). All spines arising from forefemoral, foretibial and tibial spur fuscous apically. Meso- and metatibia light brown. Fore- and hindwings hyaline, light brown, apex and costal area of forewings brownish (Fig. 2C).

**Female.** Apterous (Fig. 1B).

***Head*** (Fig. 2G). Triangular, about 1.3 times as wide as long. Compound eyes oval and large, obviously projecting outside profile of head. Juxtaocular bulges slightly exceeding dorsal margin of vertex. Ocelli smaller than male. Antennae much shorter than male. Lower front transverse 3.9 times as wide as long, dorsal and lateral margins depressed.

***Prothorax*** (Fig. 2H). 3 times as long as wide, metazone 1.5 times as long as prozone. Lateral margin with more and stronger denticulations. Several tubercules present on pronotum, especially prozone. The paramedian bulges near posterior margin more prominent. Carina on prosternum short.

***Cervix*.** As in the male.

***Metathorax*.** With cyclopean ear of DNK type.

***Forelegs*.** Forecoxa longer than metazone with anterior lobes diverging, dorsal margin with 5–6 small tubercules, ventral edge with 13–15 smaller tubercules, all tubercules with small seta, anterior and posterior of forecoxa mostly smooth (Fig. 2I). Forfemur stronger than male; forefemoral armament with four posteroventral spines; four discoidal spines, numeric patterns: 1231, the fourth discoidal spine strongly inclined toward apex; anteroventral spines with the resulting arrangement: iIiIiIiIiiI; F = 4DS/11AvS/4PvS; both genicular lobes with only one spine. Foretibial armament consisting of 5 posteroventral and 7–8 anteroventral spines; T = 7–8AvS/5PvS.

***Middle and hind legs*.** As in the male.

***Wings*.** Apterous, wing pads fused to meso- and metathorax (Fig. 1B).

***Abdomen*.** Much wider than male, fusiform. Posterior margin of 1–9 tergites and 2–6 coxosternites with several small tubercles. The lobes on the middle of tergites inconspicuous. Tergite 10 trapezoidal, longer than wide. Cerci short, slightly surpassing tergite 10.

***Coloration*.** General color brown (Fig. 1B). Vertex and juxtaocular bulge yellowish-brown (Fig. 2G). Ocelli hyaline. Clypeus with several dark spots. Tubercules on pronotum dark brown as well as denticulations on lateral margin of pronotum (Fig. 2H). Anterior surface of forecoxa with 3–4 black spots near ventral margin (Fig. 2I). Foretrochanter black ventrally. Anteroventral base of forefemur, base of tibial spur groove and the middle of them black; anteroventral base of forefemur and base of tibial spur groove black, with another irregular black spots near one-fourth basal of anteroventral margin (Fig. 2J). All spines arsing from forefemoral, foretibial and tibial spur fuscous apically. Meso- and metatibia light brown. The tubercles on abdomen fuscous (Fig. 1B).

**Figure 6.**
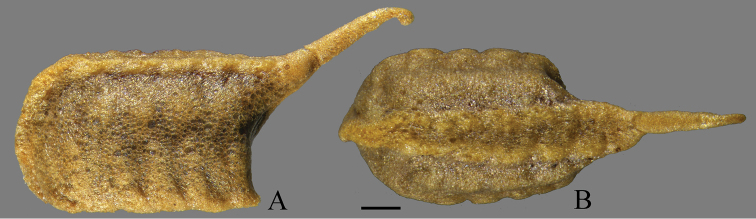
Ootheca of *Arria pura* sp. nov. **A** lateral view **B** dorsal view. Scale bar: 1 mm.

***Ootheca*** (Fig. 6). Small, rectangular, mostly trapezoid in cross-section. Residual process long, aciculiform. External wall generally russet brown, with many bubble-like structures embedded, without external coating. Ventral surface attached to complanate substrates, such as surface of leaves. Emergence area raised, openings inconspicuous. Measurements (in mm): length (without residual process), 6.1; length of residual process, 3.1; width, 3.8; thickest girth, 25.3; length of emergence area, 5.7; width of emergence area, 1.8.

#### Distribution.

China (Guizhou) (Fig. 11).

#### Etymology.

The specific name is derived from the Latin words “*pura*” (meaning pure) which refers to the forewing without any spots.

#### Remarks.

The new male species is much smaller than all other known species of *Arria*. Additionally, it can be distinguished from *A. brevifrons*, *A. cinctipes*, *A. leigongshanensis*, *A. pallida* and *A. sticta* by tegmina without any spots. *Arria pura* also differs from *A. oreophila* in having less forked RP and M.

### Arria

Taxon classificationAnimaliaMantodeaMantidae

sp. 1

7AF54A57-88BD-5254-8721-4FB5774100CE

#### Material examined.

China: 3♂♂1♀, Guizhou Prov., Dashahe National Natural Reserve, 26.V.2004, Xiang-Sheng Chen and De-Yan Ge leg. (IEGU); 3♂♂, Hunan Prov., Xiaoxi National Natural Reserve, 15–21.VIII.2016, Ying-jian Wang leg. (IEGU); 5♂♂3♀♀, Guizhou Prov., Leigongshan National Natural Reserve, 17–20.VIII.2019, Ying-jian Wang leg. (IEGU).

### Arria sticta

Taxon classificationAnimaliaMantodeaMantidae

(Zhou & Shen, 1992)

F4A20B71-BF21-5DBE-9EBB-538AC7E9C916

Palaeothespis sticta Zhou & Shen, 1992: 62–63.

#### Remarks.

The holotype is probably lost, as we did not find it in the Zhejiang Museum of Natural History where the author used to work. *Arria sticta* is similar to *A. pallida* but differs from the latter in that a dark stripe is located along the anteroventral base to the first discoidal spine of forefemur; with three dark spots on the ventral surface of forefemur.

### Sinomiopteryx

Taxon classificationAnimaliaMantodeaMantidae

Tikham, 1937

0EF58584-19F1-585A-9DB8-4D85D0686EED

Sinomiopteryx Tikham, 1937: 495; Wang and Bi 1991: 126; Wang 1993: 111; Ehrmann 2002: 319.

#### Type species.

*Sinomiopteryx grahami* Tinkham, 1937, original designation.

#### Diagnosis.

**Figure 7.**
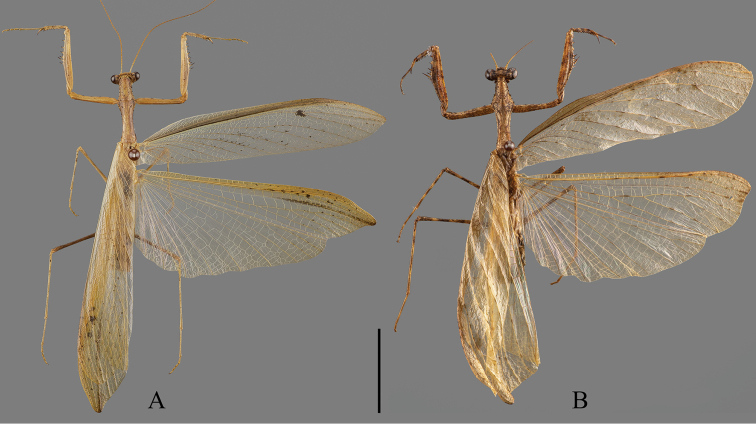
Comparition of Arriini spp., dorsal habitus **A***A.* sp.1 (IEGU: HAASP1-1) **B***S.* sp.1 (IEGU: HASSP1-1). Scale bar: 10 mm.

**Figure 8.**
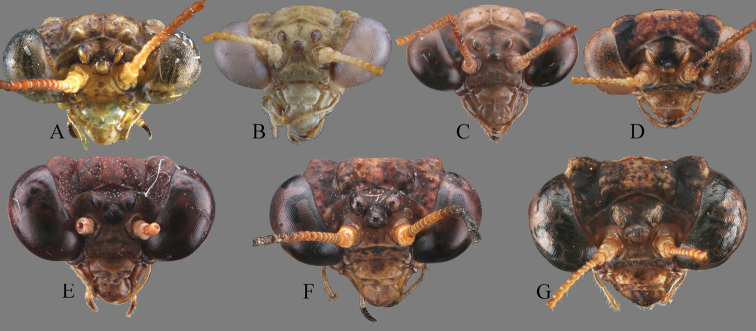
Comparison of Arriini spp., head, frontal view **A***A. leigongshanensis*, holotype **B***A. pallida* (IEGU: HAAP1) **C***A.* sp.1 (IEGU: HAASP1-1) **D***A.* sp.2 (IEGU: HAASP2-1) **E***S. guangxiensis* (IEGU: HASG1) **F***S.* sp. 1 ((IEGU: HASSP1-1) **G***S.* sp. 2 (HASSP2-1). Not to scale.

**Figure 9.**
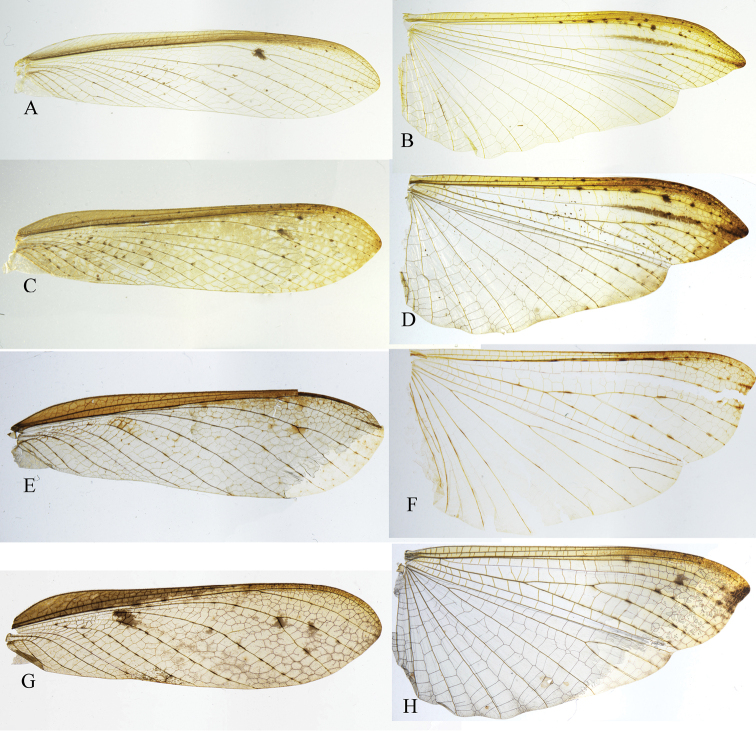
Comparison of Arriini spp., right fore- and hindwings, dorsal view **A, C, E, G** forewings **B, D–F** hindwings **A, B***A.* sp.1 (IEGU: HAASP1-1) **C, D***A.* sp.2 (IEGU: HAASP2-1) **E, F***A.* sp.2 (IEGU: HAASP2-1) **G, H***S.* sp.2 (HASSP2-1). Not to scale.

Body medium and slender (Fig. 7). Head narrowly transverse with prominent juxtaocular bulges; compound eyes broadly oval, prominent; Ocelli very large closely grouped in the male (Fig. 8E–G), minute in the female. Lower frons narrowly transverse. Antennae filiform, long in male, much shorter in female. Forefemur of males wider than in *Arria*, with 4 discoidal, 11–13 anteroventral and 4 posteroventral spines; foretibia with 8–10 anteroventral and 5–6 posteroventral spines. Pronotum short with supracoxal dilatation well marked, sparsely granulate, lateral margins with sparsely and strongly denticulate. Forewing broader than *Arria*, with rounded apex, CuA branches no more than 4, L/W ratio is 3.3–3.7, fore margin clothed with dense cilia; hindwing with truncate apex; both pairs of wings fully developed and exceeding the end of abdomen in male (Fig. 9E–H); female apterous. Sclerite L4A approximately rhomboidal, sdp thick with tip granulated, no terminal lobe (tl), right phallomere with large pia and pva. Styli far apart from each other.

### Sinomiopteryx grahami

Taxon classificationAnimaliaMantodeaMantidae

Tinkham, 1937

EDE3E45F-B149-544F-89D5-A4A5DAFBAE9B

Sinomiopteryx grahami Tinkham, 1937: 495; Wang and Bi 1991: 126; Wang 1993: 112; Ehrmann 2002: 319; Svenson 2014: 70.

#### Remarks.

Tinkham (1937) erected *Sinomiopteryx* with this male species, collected from Szechwan, Mt. Omei, Baian-Kara-Ula Range (China, Sichuan Prov., Mountain Emei), illustrated both male and female lateral habitus, and left tegmen in dorsal view. Wang and Bi (1991) and Wang (1993) illustrated the genitalia for *S. graham*, but it may have been mistaken.

### Sinomiopteryx guangxiensis

Taxon classificationAnimaliaMantodeaMantidae

Wang & Bi, 1991

6E626248-66AA-5C5D-AC3A-0A61AA186666

Sinomiopteryx guangxiensis Wang & Bi, 1991: 126; Wang 1993: 112; Ehrmann 2002: 319.

#### Material examined.

***Holotype*,** 1♂, China: Guangxi Prov., Jinxiu county, Laoshan, 24.IX.1981, Gen-tao Jin & Fu-liang Li leg. ID: 08000387 (SEM); 1♂, Guangxi Prov., Tian’e County, Longtan Nature Reserve, 18.VII.2015, Ying-jian Wang leg. (IEGU).

#### Remarks.

This species was described and illustrated by Wang and Bi in 1991 based on one male specimen from Guangxi, the holotype has had the abdomen removed, but we did not find the genitalia. Fortunately, we collected a male from Guangxi, and it perfectly fits with *S. guangxiensis*, and can be used as accurate comparative material.

### Sinomiopteryx

Taxon classificationAnimaliaMantodeaMantidae

sp. 1

C33F53CF-3F19-5C28-A05F-0D5AD77ED01E

#### Material examined.

China: 2♂, Guizhou Prov., Ziyun County, Xiaochuandong, 15.XI.2015, Ying-jian Wang leg. (IEGU); 1♂, Guizhou Prov., Ziyun County, Xiaochuandong, 15.IX.2016, Ying-jian Wang leg. (IEGU); 1♂, Guizhou Prov., Ziyun County, Xiaochuandong, 21.X.2019, Ying-jian Wang leg. (IEGU); 1♂, Guizhou prov., Duyun city, Doupengshan, 22.IX.2016, Ying-jian Wang leg. (IEGU); Hunan Prov., Xiaoxi National Natural Reserve, 20.VIII.2016, Ying-jian Wang leg. (IEGU).

### Sinomiopteryx

Taxon classificationAnimaliaMantodeaMantidae

sp. 2

7FA6B721-FB75-5580-85EF-86B216153657

#### Material examined.

China: 1♂, Yunnan Prov., Maguan County, Gulinqing town, 23.VIII.2020, Xiang-jin Liu leg. (IEGU).

**Figure 10.**
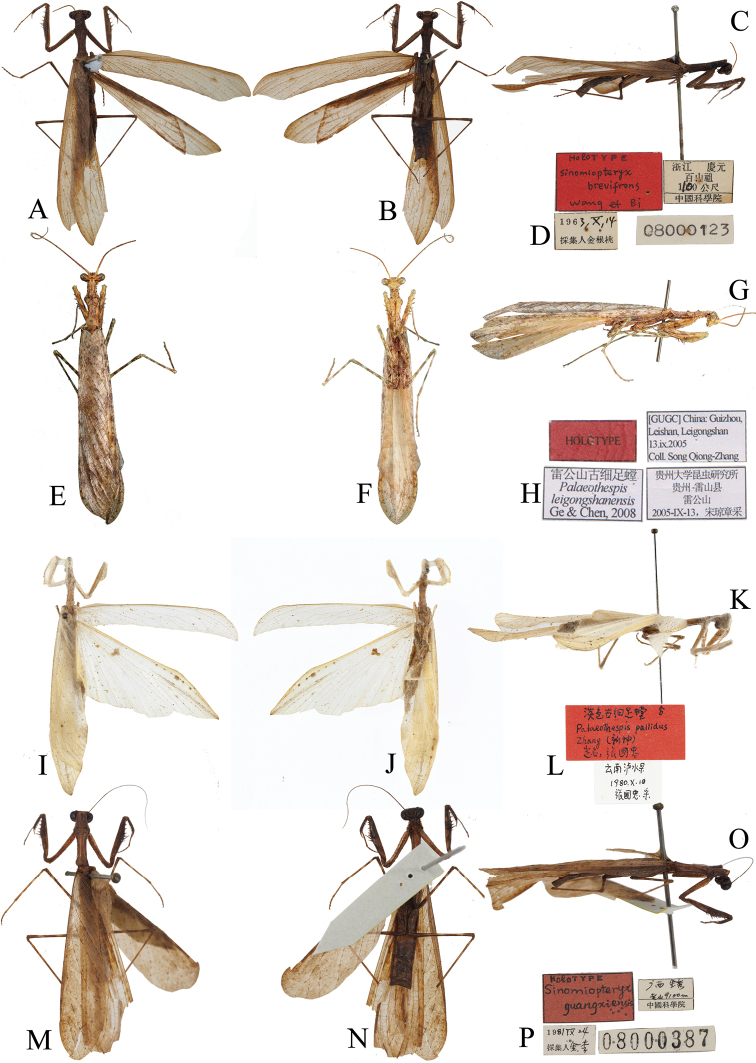
Holotypes of Arriini spp. **A, E, I, M** dorsal habitus **B, F, J, N** ventral habitus **C, G, K, O** lateral habitus **D, H, L, P** labels **A–D***A. brevifrons* comb. nov. (SEM) **E–H***A. leigongshanensis* (IEGU) **I–L***A. pallida* (NFU) M-P *S. guangxiensis* (SEM). Not to scale.

**Figure 11.**
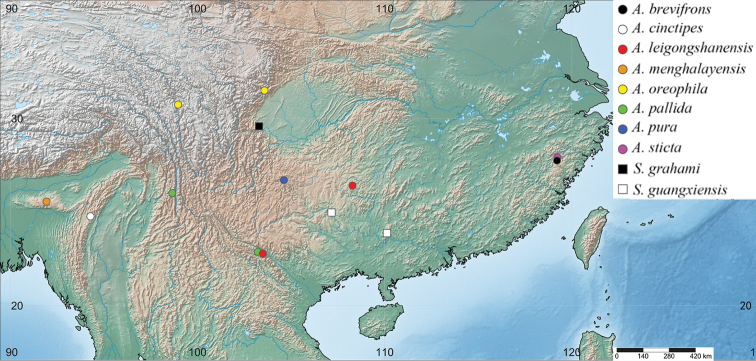
Arriini Giglio-Tos, 1919, distribution map.

## Discussion

*Arria* is superficially similar to *Sinomiopteryx*, but differs substantively in details. *Arria* can be distinguished from the latter by the following characters: (1) ventral phallomere with terminal lobe (tl) (*Sinomiopteryx* without pl); (2) styli close to each other (styli further apart in *Sinomiopteryx*) (Figs 4–5); (3) forewings narrower, L/W ratio is 4.3–5.5, CuA with five branches or more (forewings wide, L/W ratio is 3.3–3.7, CuA with four branches or less in *Sinomiopteryx*) (Fig. 9). The ocelli characters seem unstable in *Arria*, especially in *A. leigongshanensis*, they look as in *Sinomiopteryx*, but they are very large and grouped in *Sinomiopteryx* (Fig. 8). Nevertheless, identification of species may be difficult because original species descriptions are inadequate in that many features are not evaluated and included, especially the male genitalia. Besides, most females of Arriini are undescribed and difficult to distinguish from each other, unless both male and female are collected at the same time or DNA barcoding is performed. Further fieldwork is needed to uncover specimens of the rare tribe, finally allowing for its better characterization.

## Supplementary Material

XML Treatment for Arriini

XML Treatment for Arria

XML Treatment for Arria brevifrons

XML Treatment for Arria cinctipes

XML Treatment for Arria leigongshanensis

XML Treatment for Arria meghalayensis

XML Treatment for Arria oreophila

XML Treatment for Arria pallida

XML Treatment for Arria pura

XML Treatment for Arria

XML Treatment for Arria sticta

XML Treatment for Sinomiopteryx

XML Treatment for Sinomiopteryx grahami

XML Treatment for Sinomiopteryx guangxiensis

XML Treatment for Sinomiopteryx

XML Treatment for Sinomiopteryx
